# Squamous Cell Carcinoma: An Update on Diagnosis and Treatment

**DOI:** 10.5826/dpc.1003a66

**Published:** 2020-06-29

**Authors:** Andrea Combalia, Cristina Carrera

**Affiliations:** 1 Dermatology Department, University of Barcelona, Hospital Clínic de Barcelona, Spain; 2 Centro de Investigación Biomédica en Red de Enfermedades Raras (CIBERER), Instituto Carlos III, Barcelona, Spain

**Keywords:** Bowen disease, squamous cell carcinoma, actinic keratosis, cancerization field, dermoscopy, confocal microscopy, staging, treatment

## Abstract

Squamous cell carcinoma (SCC) accounts for most nonmelanoma skin cancer–related metastatic disease and deaths. Histopathology and correct surgical excision remain the gold standard for the diagnosis and treatment of SCC; however, new diagnostic imaging techniques such as dermoscopy and reflectance confocal microscopy have increased the diagnostic accuracy in terms of early recognition, better differential diagnosis, more precise selection of areas to biopsy, and noninvasive monitoring of treatments. The therapeutic intervention in patients with severe actinic damage and multiple in situ/low-risk SCC, and the development of innovative treatments such as epidermal growth factor receptor inhibitors and immune checkpoint inhibitors for locally advanced and metastatic SCC, are improving considerably the approach to the disease. This review summarizes the up-to-date knowledge in the field of detection, treatment, and monitoring of cutaneous SCC.

## Introduction

Squamous cell carcinoma (SCC) is the second most common cutaneous malignancy after basal cell carcinoma, with an increasing incidence worldwide [1]. Although many factors can increase the risk for SCC, cumulative sun exposure, especially in childhood and youth, is of greatest importance. Moreover, in recent years, immunosuppression, including that associated with organ transplantation, has emerged as an increasingly important contributor to tumorigenesis [2,3], and the arousal of SCC in areas of chronic inflammation must also be kept in mind.

SCC accounts for most nonmelanoma skin cancer–related metastatic disease; therefore, recognition and treatment of early SCC is important for the prevention of neoplastic progression. Although histopathology and surgical excision remain the gold standard for the diagnosis and treatment of SCC, new diagnostic imaging techniques such as dermoscopy and reflectance confocal microscopy (RCM) are increasing the diagnostic accuracy of these keratinizing neoplasms, allowing better recognition and a more precise selection of suspicious areas to biopsy, and provide a noninvasive, accurate way to monitor treatments. Moreover, the therapeutic intervention on the cancerization field in patients with severe actinic damage and multiple in situ/low-risk SCC, and the development of innovative treatments such as epidermal growth factor receptor inhibitors and immune checkpoint inhibitors for locally advanced and metastatic SCC, are improving considerably the approach to the disease.

## Update on the Diagnosis

### Clinical and Dermoscopic Presentation

**Figure 1:**
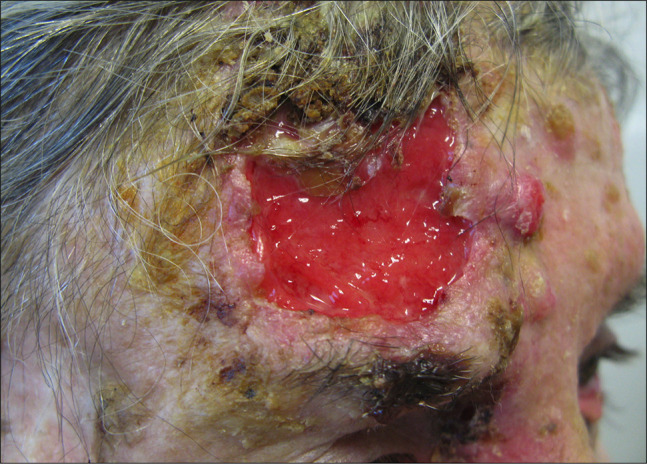
Advanced and recurrent squamous cell carcinoma with nodal involvement in an elderly woman with dementia and chronic myeloid leukemia. Large ulcerative and keratotic tumor with numerous actinic keratosis and satellite nodular lesions on the surrounding skin. She presented with locoregional lymph node involvement on parotid homolateral region.

**Figure 2:**
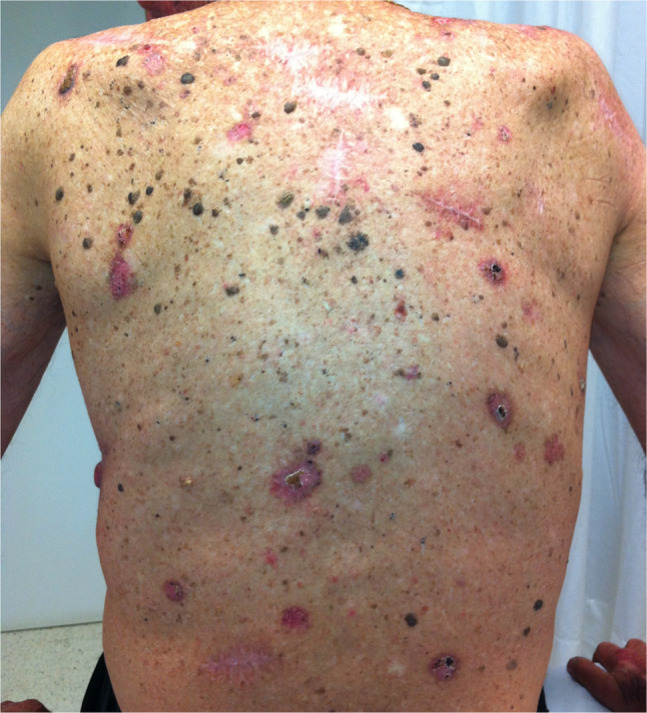
Multiple skin cancers, mainly squamous cell carcinomas, in a solid organ transplant patient.

**Figure 3:**
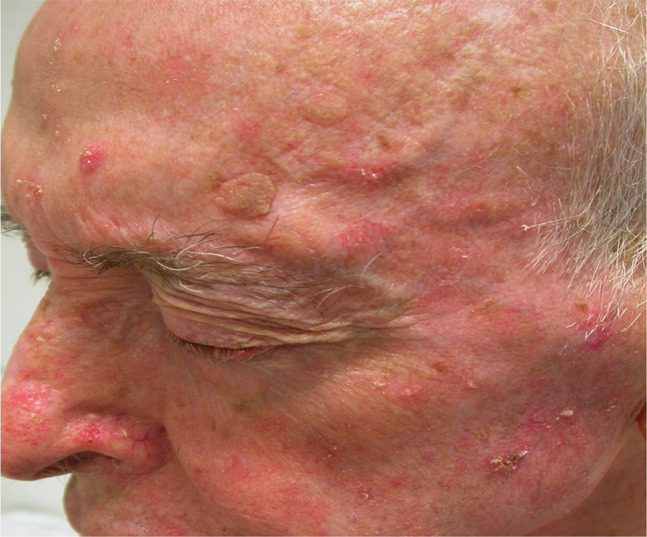
Example of cancerization field. Multiple actinic keratoses in a patient with marked sun-damaged skin.

**Figure 4:**
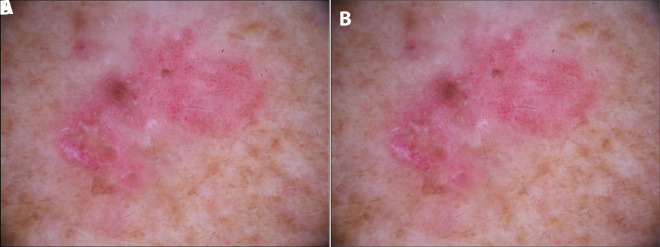
(A) Clinical image of Bowen disease. (B) Dermoscopic image of Bowen disease where clusters of glomerular and dotted vessels and whitish scales can be identified.

**Figure 5:**
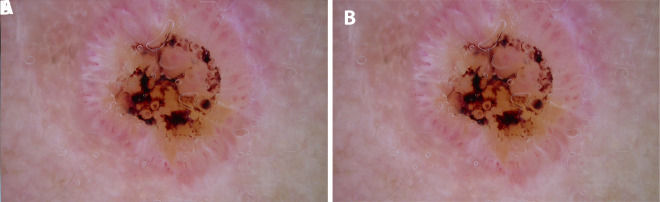
(A) Clinical image of a well-differentiated squamous cell carcinoma (keratoacanthoma-like) on the nose of a patient with sun-damaged skin. (B) Dermoscopy shows a symmetric lesion with a central ulceration, surrounded by hairpin vessels with a whitish halo in a crown distribution.

A presumptive diagnosis of SCC is based on the physician’s interpretation of clinical information, including appearance and morphology, anatomic location, and patient-reported history. While the most frequent clinical presentation of SCC in situ is an erythematous scaly patch or slightly elevated plaque, which is barely noticed by the patients, invasive SCC is often ulcerated and can be patchy, papulonodular, papillomatous, or exophytic (Figures 1–5).

Although histopathology remains the gold standard for the diagnosis of SCC, some noninvasive optical technologies such as dermoscopy and RCM have recently been applied in an attempt to enhance clinical diagnosis accuracy and to obtain an in vivo characterization of the tumor [4].

**Figure 6:**
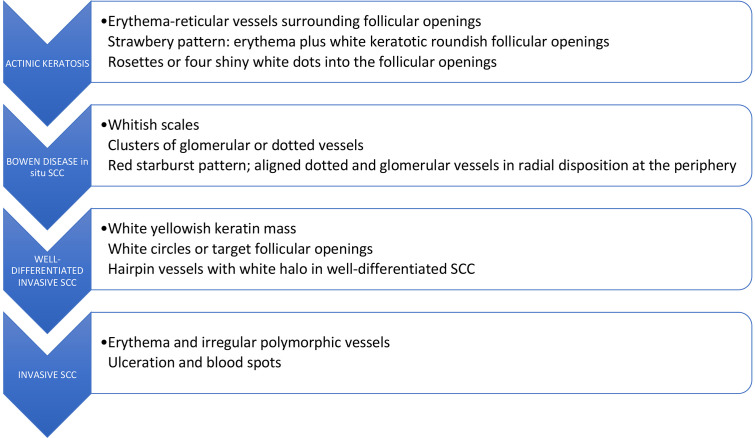
Dermoscopic features found in the progression model of actinic keratosis into squamous cell carcinoma (SCC) in situ and invasive based on the article of Zalaudek et al [6].

Dermoscopy is a noninvasive diagnostic technique that allows in vivo evaluation of colors and microstructures of the epidermis, the dermoepidermal junction, and the papillary dermis at 10-fold magnification [1], which improves diagnostic accuracy for SCC compared to inspection by the unaided eye [5]. Although there is some overlap between dermoscopic features of actinic keratosis (AK), in situ SCC (Bowen disease), and microinvasive SCC, there are also some important indicators that can assist in the diagnosis of SCC and its subsequent management [6]. Table 1 and Figure 6 summarize the main dermoscopic findings of the spectrum of AK and SCC [7].

In situ SCC (Bowen disease) is characterized by yellowish white opaque scales and clusters of 2 types of roundish vascular pattern: small dotted vessels and glomerular or coiled vessels. Both patterns often appear within the same lesion and are distributed in small, densely packed clusters or groups [1]. In pigmented Bowen disease, other dermoscopic features are represented by small brown globules, which are regularly packed or aligned in a patchy distribution, and by gray to brown homogeneous pigmentation. White circles are a specific feature of early SCC and actinic keratosis. They are white structures within the hair follicle that might present a ring-like or targetoid appearance due to the white yellowish keratotic plug in the center of the follicular opening and white halo surrounding it.

When in situ SCC progresses to microinvasive SCC, the lesion thickens clinically, and hairpin and/or linear-irregular vessels appear in dermoscopy examination, occasionally giving a red starburst-like image on dermoscopy. In addition to typical vessel morphology, a keratotic center and ulceration might be seen. Invasive SCC presents more polymorphic vessels such as linear irregular, hairpin and grouped glomerular/dotted vessels over a whitish background with a central mass of keratin or ulceration [1]. Such dermoscopic features reflect a “vertical” growth phase (dermal invasion) [6].

The combination of clustered dotted/glomerular vascular patterns and hyperkeratosis, seen as discrete yellow scales, has previously been shown to achieve 98% diagnostic probability for SCC [7]. However, the adherent surface scale often obscures the underlying morphological features, meaning that vessels may be better visualized at the periphery of the lesion. Moreover, the absence of these vessels will not exclude the presence of an in situ SCC. Furthermore, the same pattern described for in situ SCC can be found in eczematous lesions, showing the necessity of integrating clinical examination with dermoscopic features and other imaging techniques.

### Other In Vivo Imaging Techniques

#### Reflectance Confocal Microscopy

RCM is a noninvasive technique for in vivo imaging of the skin with a cellular-level resolution (0.5–1.0 mm in the lateral dimension and 4–5 mm the axial) [8] that uses near-infrared laser light at 830 nm. The imaging depth is in relation to the wavelength of the laser light used, but limited to 200–300 mm, which corresponds to the papillary dermis in normal skin [8]. This technique reproduces horizontal images of the skin in shades of gray, with a resolution comparable with that of conventional histology. It is painless and harmless; it allows the evaluation of a larger area of skin, the mapping of a whole tumor and margins, and the imaging of exactly the same location over time; and it does not induce any kind of skin damage or inflammatory response [9]. In the context of keratinizing tumors, if RCM is available in referral centers, it can be used routinely for several purposes: diagnosing the wide spectrum of nonmelanoma skin cancer-AKs, demarcation of surgical margins of tumors, differential diagnosis from other tumors on sun-damaged skin, and monitoring both invasive and noninvasive therapies such as topical imiquimod, 5-fluorouracil, photodynamic therapy, laser therapy, or cryotherapy. However, there is a clear limitation to the ability of the microscope to evaluate the thickest components of the tumors, as occurs in some cases of SCC in which hyperkeratosis predominates. The presence of hyperkeratotic surface on the lesion can make the RCM examination challenging, as the epidermal layers might be poorly visualized due to limited RCM laser penetration [9,10].

Moreover, early recognition of SCC by RCM is difficult, and clear differentiation between AK and in situ SCC or between in situ and invasive SCC remains a challenge, as keratinocyte atypia is considered in both the most relevant parameter [11,12]. AKs on RCM are characterized by individual, highly refractile, round nucleated cells in the stratum corneum (which indicates parakeratosis) and an increase of thickness of stratum corneum seen as refractile amorphous material (hyperkeratosis) [13]. However, the presence of architectural disarray (severe disarranged epidermal pattern in which the honeycomb pattern is no longer visible) in the stratum granulosum in combination with architectural disarray in the spinous layer and/or tumor nests in the dermis (aggregates of atypical keratinocytes) has been reported as the main RCM features to distinguish SCC from AK [14]. Moreover, the presence of round nucleated cells at the spinous-granular layers (which means parakeratosis and dyskeratotic cells) and round blood vessels traversing through the dermal papilla are the key features of SCC on RCM [10,15]. The roundish vessels, which correspond to the “glomerular vessels” typically seen by dermoscopy, can be found in SCC by RCM even when they are not clearly visible with other imaging technologies. Table 2 summarizes the features of AK and SCC by RCM [9].

#### High-Frequency Ultrasonography and Doppler Mode

High-frequency ultrasonography (HFUS) is a fast and accessible, noninvasive, convenient, practical, and safe dermatological diagnostic imaging examination that is now widely used in skin cancer. It allows real-time visual information with high diagnostic value and provides the physician with an extra hand in everyday practice. HFUS uses frequencies of approximately 20 MHz, which are dedicated to depicting the skin and allow scanning of the whole skin (epidermis, papillary, reticular dermis, blood vessels, and upper parts of subcutaneous tissue—depending on the localization). As the ultrasound probe increases in frequency, its resolution increases but penetration decreases. Used in conjunction with clinical and/or dermoscopic examination of suspected skin cancer, HFUS may offer additional diagnostic information compared to other technologies. In fact, Wortsman et al, in a large retrospective study of 4,338 ultrasonographic skin examinations, showed that the addition of HFUS increased the correctness of clinical diagnosis from 73% to 97% [16].

In HFUS, tumor depth is ascertained with B-mode, and Doppler blood flow technologies permit the measurement of tumor neovascularity and the mapping of vascular structures. SCC is usually seen as a hypoechogenic mass in relation to the surrounding tissue, without clear specific features that allow the differential diagnosis from other nonmelanoma skin cancer or other skin lesions. Sometimes the inflammation and the scar tissue beneath or surrounding the lesion can be isoechoic to the tumor and lead to misjudgment of borders. Moreover, the hyperkeratotic scale characteristic of some SCC might also interfere in the image of the tumor. In fact, to study the lesions that have severe crusting or keratinization such as some SCC, it is recommended to remove the scale or crust, since they cause attenuation of the sound beam, reducing the accuracy of the test. Accordingly, Marmur et al pointed out that, owing to the SCC characteristic of generally presenting hyperkeratosis and to the higher inflammatory process associated, the tumor area could be overestimated in some cases when evaluated by ultrasound [17]. For this reason, HFUS must always be considered a complementary diagnostic technique in SCC.

However, HFUS is useful for determining the local aggressiveness of the tumor. SCC is more likely to invade soft tissues, cartilage, and adjacent bone than other nonmelanoma skin cancer, and this can be assessed with ultrasonography. In fact, currently, the main clinical use of HFUS in SCC regards preoperative assessment of the invasion in critical areas such as the face or the scalp, as HFUS gives a clear picture of the size and depth of the tumor, which is particularly important in planning the extent of the surgery.

#### Optical Coherence Tomography

Optical coherence tomography (OCT) is an emerging technology that uses infrared light for performing high-resolution, cross-sectional imaging. OCT is analogous to ultrasound imaging, except that it uses light instead of sound [1] and it magnifies the surface of a skin lesion using near-infrared light. Used in conjunction with clinical or dermoscopic examination of suspected skin cancer, or both, OCT may offer additional diagnostic information. As occurs in HFUS, the method is useful for preoperative evaluation of the tumor size in patients with SCC, as it provides a real-time imaging of nonmelanoma skin cancer to a depth of approximately 1.5 mm [18]. However, its specificity is low and there are no pathognomonic features of SCC under OCT examination.

### Diagnosis Confirmation, Staging, and Follow-up

Diagnosis is routinely confirmed by biopsy, and histological examination will differentiate between in situ and invasive SCC and detect aggressive histopathological growth patterns. However, conventional histopathological examination is also undergoing some changes, as new imaging techniques are emerging and beginning to replace the typical fixation, processing, and staining methods. This is the case of ex vivo fluorescence and RCM, which allows a rapid microscopic examination of freshly excised unfixed tissue directly in the surgery room during Mohs surgery after a few seconds of immersion in a fluorescence media [19]. This optical imaging modality uses the inherent light-scattering properties of the different components of the tissue and generates optical images similar to H&E-stained tissue sections obtained by cutting frozen or fixed tissues [20].

Under conventional histopathology or ex vivo RCM, features such as the degree of differentiation, aggressive histological subtypes (acantholytic, adenosquamous, and carcinosarcomatous), depth greater than 2 mm (measured from the granular layer of the adjacent intact epidermis), Clark level IV or greater, and presence of perineural and/or angiolymphatic invasion classify the lesion as high risk [2]. The differential diagnosis between SCC subtypes is mandatory, as it will further determine the therapeutic approach and follow-up of the tumor.

Once an invasive/aggressive SCC is diagnosed, it must be staged as the risk for metastasis is reported to be approximately 4% [21], and even 2 to 3 times higher in immunosuppressed individuals [22]. Locoregional invasion can be assessed by HFUS or OCT in some cases as mentioned above. However, additional imaging techniques such as computed tomography (CT) or magnetic resonance imaging (MRI) are frequently required to assess the depth of invasion in critical areas such as the face or the scalp, as SCC is more likely to invade soft tissues, cartilage, and adjacent bone than other nonmelanoma skin cancer. Cutaneous in-transit regional lymph node metastases are the most common metastatic presentation; therefore, clinical assessment of regional lymph nodes must be always included in the physical examination and supported by complementary imaging techniques such as ultrasonography and positron emission computed tomography (PET/CT).

Staging of the tumor is classically performed according to the TNM (tumor, node, and metastasis) American Joint Committee on Cancer (AJCC) criteria [23]. However, the latest guidelines of care for the management of SCC suggest that the staging of localized SCC in clinical practice might be done using the current National Comprehensive Cancer Center (NCCN) risk stratification, as they include both clinical and pathological parameters, and suggest therapeutic approaches for each stage of the tumor [24], and that the Brigham and Women’s Hospital (BWH) tumor classification system [25] might be chosen to evaluate the prognosis and clinical outcome of the patients diagnosed with SCC, as it provides a more accurate method of prognostication.

In all the staging scales there are several clinical-pathological markers of high-risk SCC such as tumor size (which can vary depending on the location), Breslow or tumor depth, perineural and lymph/vascular invasion, histological differentiation of the tumor (despite not being included in the latest AJCC classification), and the immune status of the patient (worse prognosis is seen in immunocompromised patients). When the patient fulfills 2 or more high-risk markers, further staging by sentinel lymph node biopsy (SLNB) is recommended, as about 20% of these patients will present positive SLNB. However, the impact on the overall survival or disease-free survival has still not been established [25,26].

Follow-up of patients must be individualized and determined both by the clinical history of the individual and the subtype of the SCC; however, screening for new primary skin cancers should be performed at least once per year, adjusting frequency on the basis of individual patient risk.

## Update on the Treatment

The recently published guidelines for SCC [2] resume the evidence-based recommendations for clinical treatment and management of patients with SCC.

### Surgical Treatment

Surgical excision is still the gold standard and includes conventional and Mohs surgery [26]. Conventional excision must ensure complete removal and therefore include a margin of clinically normal-appearing skin around the tumor and surrounding erythema. Clinical margins can be assessed prior to surgery by imaging techniques such as dermoscopy, RCM, HFUS, or OCT, which decrease the rates of incomplete excision and affected margins. NCCN guidelines recommend 4- to 6-mm clinical margins for standard excision of low-risk SCC [24], whereas Mohs surgery is recommended in high-risk SCC, SCC in immunocompromised patients, or “special-site SCC” such as head and neck, where tissue conservation is important [27]. The criteria for appropriate use of Mohs surgery was recently updated by a combined task force of the American Academy of Dermatology, the American College of Mohs Surgery, the American Society for Dermatologic Surgery Association, and the American Society for Mohs Surgery [27].

### Nonsurgical Treatment

#### Low-Risk SCC

If surgical therapy is not feasible or elected, nonsurgical local approaches may be considered [2]. For in situ or low-risk SCC, the physician can choose photodynamic therapy (a 2-step method consisting of topical application of a photosensitizer, either 5-aminolevulinic acid or methylaminolevulinate, followed by 1 to several hours of incubation by light irradiation, typically with a blue, red, or broadband light source), or topical therapy with imiquimod (3.75%–5%), or 5-fluorouracil. These modalities not only treat the tumor, but also have an effect on the cancerization field if applied in a larger area.

Nonsurgical ablative modalities are also accepted in some cases in which surgery is not feasible, contraindicated, or not preferred by the patient. These include laser ablation (CO2, erbium), electrocoagulation, and cryosurgery. However, given the lack of histological margin control with these approaches, the recurrence rate of SCC is higher [28]. Primary local radiation can also be used in special situations when other therapies are contraindicated or impractical [2,29].

#### High-Risk SCC and Metastatic Disease

High-risk SCC should always be surgically excised, preferably by Mohs technique; however, adjuvant radiation therapy to the local tumor site following surgical treatment may be considered in primary SCC concerning perineural invasion or at high risk for regional or distant metastasis.

Regarding locally advanced and metastatic SCC, treatment is based on the extent of disease. If lymph nodes are involved, dissection must be performed whenever possible, and adjuvant radiation with or without concurrent systemic therapy must be considered [2]. Systemic therapies such as capecitabine or epidermal growth factor receptor inhibitors (cetuximab, panitumumab) [30,31] have demonstrated efficacy in patients with advanced, unresectable SCC. Based on the high mutational loads of SCC, the well-known infiltration with lymphocytes, and programmed death ligand 1 (PD-L1) expression, there is a promising utility in treating SCC with the immune checkpoint inhibitors such as pembrolizumab [32]. Recently the first anti-PD1 drug cemiplimab was approved by the US Food and Drug Administration (September 2018) and by the European Medicines Agency (July 2019) after having demonstrated responses in about 50% of advanced or metastatic SCC [33].

## Conclusions

The main advances in SCC diagnosis are in the field of noninvasive imaging techniques. While histopathology remains the gold standard, noninvasive imaging techniques such as dermoscopy and RCM have recently been applied not only to enhance clinical diagnostic accuracy, but also to achieve an in vivo microscopic characterization of the tumors and margins previous to biopsy and surgery. The role of Doppler ultrasonography in evaluating the deep invasive components and lymph node involvement is unquestionable. As a result, the staging, management decisions, and treatment monitoring of the SCC is continuously improving thanks to the development of skin imaging techniques. Moreover, the current concept of cancerization field and the development of several nonsurgical treatment modalities (imiquimod, 5-fluorouracil, ablative laser plus photodynamic therapy) have provided a wider range of therapies for low-risk SCC and actinic keratosis. On the other hand, the new available systemic therapies (immune checkpoint inhibitors and target therapies with anti-epidermal growth factor receptor) for locally advanced and metastatic SCC are improving the management and hopefully the outcome of SCC.

## Figures and Tables

**Table 1:** Dermoscopic Features of Actinic Keratosis and Squamous Cell Carcinoma Spectrum

Dermoscopic Criteria	Description
Red color: vessels	
Erythema	Structureless pale red areas without any recognizable areas
Red pseudonetwork	Structureless red areas intermingled with small roundish white areas that correspond to follicular infundibulum
Red starburst	Radial arranged structureless red lines or hairpin vessels that surround a yellow to white structureless scaly center and that have an overall starburst appearance
Dotted vessels	Tiny red dots densely aligned next to each other
Glomerular vessels	Variation on theme of dotted vessels, they are larger than dotted vessels, have convoluted morphology, and are often distributed in clusters
Hairpin vessels	Vascular loops sometimes twisted and bent, usually surrounded by a whitish halo when seen in keratinizing tumors
Linear irregular vessels	Linear or slightly curved; irregularly shaped, sized, and distributed red structures
Whitish yellow structures	
White circles (targetoid hair follicle)	Roundish structures of varying size composed of a white yellowish to light brown structureless center and a white outer structureless rim; this pattern corresponds to keratotic plugs within follicular openings of skin
Rosettes	Four closely aggregated white, small dots corresponding to follicular opening and resembling a 4-leaf clover; if one imagines connecting 4 dots with one line, the geometric figure of a rhombus can be formed
White structureless areas	The absence of any structure; they may cover large areas of the tumor and may be associated with large targetoid hair follicles
Yellow to light brown opaque scales	Yellow to light brown opaque structureless areas with a scaly or keratotic aspect which do not cover large areas of the tumor surface
Keratin mass	Centrally located, amorphous, yellow-white to light brown areas without any recognizable structure
Red-brown structures	
Erosions	Small and irregularly distributed orange to red to red-brown structureless areas; they correspond to superficial hemorrhages and are usually associated with yellow opaque structures
Ulceration	Large irregularly shaped or roundish areas of dull red or red-brown structureless color

**Table 2:** Reflectance Confocal Microscopy (RCM) Findings in Actinic Keratosis and Squamous Cell Carcinoma and Their Histopathological Correlations [9,15]

Level/Features	Description	Histopathological Correlation
Stratum corneum		
Scale	White refractile amorphous material	Increase of thickness of stratum corneum
Parakeratosis	Individual highly refractile round cells with a hyporefractile visible nucleus	Parakeratotic cells
Epidermis spinous stratum		
Atypical honeycomb pattern	Irregular size and shape of cells with variable thickness and brightness of the lines	Abnormal pattern of the spinous layers
Targetoid cells	Cells with a refractile nucleus, hyporefractile periphery, and refractile membrane (target-like)	Apoptotic large cells
Epidermis spinous-granular layers		
Disarranged and disrupted epidermal pattern	Severe architectural disarray in which the honeycomb pattern is no longer visible	Epidermis spinous-granular layers
Epidermis (full thickness)		
Atypical honeycomb pattern	Irregular honeycomb also affecting the stratum granulosum	Cellular atypia affecting the complete epidermal layers
Dermoepidermal junction		
Thickened papillae	Enhanced honeycomb surrounding the dermal papillae	Atypical hyperplasia of the basal epidermal layer surrounding the dermal papilla
Dermis		
Dermal elastosis	Curled and clumped fibers in upper dermis and slightly small dilated vessels	Dermal elastosis
Round blood vessels	Dilated blood vessels within the dermal papillae that run perpendicular to the horizontal RCM plane of imaging (S-shaped vessels)	Neovascularization
Irregular and hairpin vessels	Atypical and marked dilated loop vessels surrounding refractile tumor nests	Neovascularization
